# Association between the risk of heart failure hospitalization and end-stage renal disease with digoxin usage in patients with cardiorenal syndrome: A population-based study

**DOI:** 10.3389/fpubh.2022.1074017

**Published:** 2023-01-17

**Authors:** Kai-Ting Chang, Hsuan-Fu Kuo, Yu-Han Chang, Yu-Tsang Wang, Lii-Jia Yang, Sheng-Wen Niu, I-Ching Kuo, Yumay Chen, Zhi-Hong Wen, Chi-Chih Hung, Jer-Ming Chang, Hugo Y.-H Lin

**Affiliations:** ^1^Department of Pediatrics, Kaohsiung Municipal Ta-Tung Hospital, Kaohsiung, Taiwan; ^2^Department of Pediatrics, Kaohsiung Medical University Hospital, Kaohsiung Medical University, Kaohsiung, Taiwan; ^3^Department of Internal Medicine, Kaohsiung Municipal Ta-Tung Hospital, Kaohsiung, Taiwan; ^4^Division of Cardiology, Department of Internal Medicine, Kaohsiung Medical University Hospital, Kaohsiung, Taiwan; ^5^Management Office, Kaohsiung Municipal Ta-Tung Hospital, Kaohsiung, Taiwan; ^6^Division of Nephrology, Department of Internal Medicine, Kaohsiung Medical University Hospital, Kaohsiung, Taiwan; ^7^School of Medicine, University of California, Irvine, Irvine, CA, United States; ^8^Department of Marine Biotechnology and Resources, National Sun Yat-sen University, Kaohsiung, Taiwan; ^9^Department of Medicine, College of Medicine, Kaohsiung Medical University, Kaohsiung, Taiwan

**Keywords:** digoxin, cardiorenal syndrome, CKD, ESRD, heart failure

## Abstract

**Background:**

The management of the coexistence of heart disease and kidney disease is increasingly challenging for clinicians. Chronic kidney disease (CKD) is not only a prevalent comorbidity of patients with heart failure but has also been identified as a noteworthy risk factor for all-cause mortality and poor clinical outcomes. Digoxin is one of the commonest treatments for heart disease. There are few trials investigating the role of digoxin in patients with cardiorenal syndrome (CRS). This study aims to examine the association between digoxin usage and clinical outcomes in patients with CRS in a nationwide cohort.

**Method:**

We conducted a population-based study that included 705 digoxin users with CRS; each patient was age, sex, comorbidities, and medications matched with three non-users who were randomly selected from the CRS population. Cox proportional hazards regression analysis was conducted to estimate the effects of digoxin on the incidence of all-cause mortality, congestive heart failure (CHF) hospitalization, coronary artery disease (CAD) hospitalization, and end-stage renal disease (ESRD).

**Results:**

The all-cause mortality rate was significantly higher in digoxin users than in non-users (adjusted hazard ratio [aHR] = 1.26; 95% confidence interval [CI] = 1.09–1.46, *p* = *0.002*). In a subgroup analysis, there was significantly high mortality in the *0.26–0.75* defined daily dose (DDD) subgroup of digoxin users (aHR = 1.49; 95% CI = 1.23–1.82, *p*<*0.001*). Thus, the *p for trend* was *0.013*. With digoxin prescription, the CHF hospitalization was significantly higher [subdistribution HR (sHR) = 1.17; 95% CI = 1.05–1.30, *p* = *0.004*], especially in the >*0.75 DDD* subgroup (sHR = 1.19; 95% CI = 1.01–1.41, *p* = *0.046; p for trend* = *0.006*). The digoxin usage lowered the coronary artery disease (CAD) hospitalization in the > *0.75 DDD* subgroup (sHR = 0.79; 95% CI = 0.63–0.99, *p* = *0.048*). In renal function progression, more patients with CRS entered ESRD with digoxin usage (sHR = 1.34; 95% CI = 1.16–1.54, *p*<*0.001*). There was a significantly greater incidence of ESRD in the < *0.26 DDD* and *0.26*–*0.75 DDD* subgroups of digoxin users (sHR = 1.32; 95% CI = 1.06–1.66, *p* = *0.015*; sHR = 1.44; 95% CI = 1.18–1.75; *p for trend*<*0.001*).

**Conclusion:**

Digoxin should be prescribed with caution to patients with CRS.

## Introduction

The prevalence rates of heart failure in the United States and Europe are estimated to be up to 14% (1). Heart failure is not uncommon in East Asia, and the annual incidence is 22 per 1,000 population in elderly in Taiwan (2). CKD is highly co-prevalent with heart failure in a position of 50% due to the interconnection between heart diseases and kidney diseases (3). The reduced estimated glomerular filtration rate (eGFR) is an independent risk factor for worse outcomes in patients with heart failure. The risk of cardiovascular death and hospitalization of patients with heart failure is raised with advanced CKD stages (aHR, 1.54 for 45–60 ml/min per 1.73 m^2^ and 1.86 for < 45 ml/min per 1.73 m^2^) (4). The all-cause mortality (hazard ratio of 1.50 for 45–60 ml/min per 1.73 m^2^, and 1.91, for < 45 ml/min per 1.73 m^2^) increased stepwise with the decline of eGFR (4).

Cardiorenal syndrome (CRS) encloses the various interactions between the heart and the kidneys (5). The CRS are divided into five types depending on the direction of action and whether the triggering injury is acute or chronic (3). It is the result of complex pathophysiologic processes. There are currently three fundamental pathophysiology thoughts to commit to the development and progression of heart and kidney interactions. First, hemodynamic changes due to decreased left ventricular ejection fraction and/or altered venous return (6, 7). The renal function is dependent on renal plasma flow and filtration fraction. With inconsistent renal perfusion owing to impaired cardiac output, renal autoregulation will be disrupted (6). When the kidney receives lower than 25% cardiac output, this hypoperfusion will trigger the baroreceptors of the kidney, and the renin–angiotensin–aldosterone system (RAAS) will be activated, inducing renal vasoconstriction, which ultimately leads to renal injuries (8). Second, the increase of central venous pressure with or without right atrial pressure alteration stimulates sympathetic nerve activation and dysregulates the neuro-hormonal axis of the heart and the kidney (9, 10). Overactivation of the sympathetic nervous system will exacerbate the heart failure progression (11). Third, the other factors that contribute to the worsening of the heart and the kidney include the immune system, metabolic disorders (including diabetes, metabolic syndrome, and obesity), oxidative stress, uremic molecules, and epigenetic factors (12). Based on the initial pathology, there are two main CRS groups: cardiorenal and reno-cardiac, which are further split into five forms of CRS, and the majority are type 2 and type 4 CRS (13). Each type of CRS has crosstalk, and each one has the potential to develop into a cycle that exacerbates both the primary and secondary conditions.

The concur of CKD with heart disease in CRS obscures the management of heart failure. Treatment to alleviate congestive symptoms of heart failure is restricted by an additional decrease in renal function (14). Then, the cyclical nature of CRS occurs in around one-fifth of late-stage patients with CKD, which often complicates its management (15). Currently, the main treatment of CRS is correcting traditional and non-traditional cardiovascular risk factors and preventing CKD progression. Guidelines for the management of heart failure in the general population may not apply entirely to those with CRS since such patients were often excluded from most of the randomized controlled trials. Although the development of new classes of medications for heart failure, including sodium–glucose cotransporter-2 inhibitor (SGLT2i) and angiotensin receptor neprilysin inhibitor (ARNI), digoxin is still one of the treatments of choice for cardiovascular disease. According to the Kidney Disease Outcomes Quality Initiative (KDOQI) guideline, digoxin is included in its ESRD cardiovascular disease guidelines for the treatment of CHF (16). However, in the study of Chan et al., digoxin use among patients with ESRD on hemodialysis was associated with increased mortality (17). There are few studies that have verified the safety of digoxin in patients with CRS, who are prone to worsen renal function that may directly mediate the efficacy and toxicity of the drug. Notably, 85% of administered digoxin is excreted renally. The risk of toxicity from this narrow therapeutic window may be greatly elevated in patients with renal dysfunction.

We designed and conducted an analysis using data from the Taiwanese National Health Insurance Research Database (NHIRD), which is one of the largest nationwide databases in the world (18). This study aims to investigate the relationship between digoxin usage and clinical outcomes in patients with CRS.

## Methods

### Study population

The Taiwanese National Health Insurance (NHI) system is a compulsory national insurance that covers 99% of Taiwan's 23.74 million residents and contracts with 97% of Taiwanese healthcare providers (18). The Taiwanese National Health Insurance Research Database (NHIRD), which compiles claim data from the National Health Insurance Research Institutes (NHRI), has been de-identified for research and made public for the first time since 2000. The NHIRD exemplifies a population-level data source where researchers have access to robust information, including details of inpatient and ambulatory care, prescriptions dispensed at pharmacies, and health service utilization of medical facilities (19). The diseases diagnosed are coded according to the International Classification of Diseases, Ninth Revision, Clinical Modification (ICD-9-CM). This cohort study was approved by the Institutional Review Board of Kaohsiung Medical University (KMUHIRB-EXEMPT(II)-20160025) with IRB exemption due to no more than minimal risk, and all of these research procedures fit within the exemption categories in the KMUH IRB regulations.

### Design and study participants

**Figure 1:**
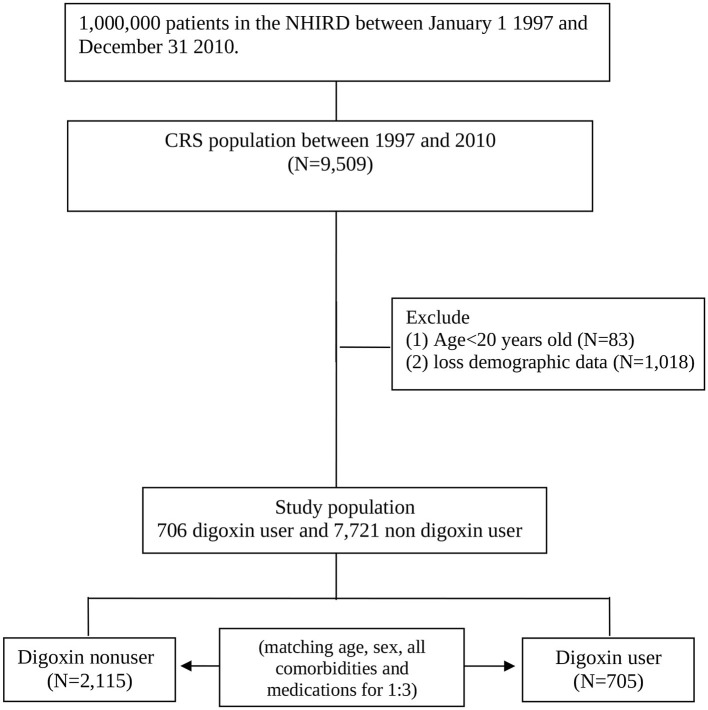
Flow diagram of the study population.

We conducted a retrospective cohort study. Patients with CRS type 2 and type 4 coexisting with the CHF diagnoses (ICD-9-CM: 428) and one of the CKD diagnoses (ICD-9-CM codes 581–587) between 1 January 1997 and 31 December 2010 according to the NHIRD records. We identified these patients as patients with CRS when they have both the above-stated diagnoses on the same date of hospitalization or at the same date of outpatient. We identified the date of the first CRS diagnosis as the index date. We matched according to sex, age, all comorbidities, and medications with a ratio of one to three for the study cohort with the definition of digoxin usage more than 90 days and comparison cohort randomly selected patients within the cohort without taking digoxin. The index year was defined as the year of CRS diagnosis. Age was calculated from the date of birth to the date of CRS diagnosis. The follow-up period started from the date of entering the study cohort to the date of the clinical event, censoring, or 31 December 2010 (Figure 1). Digoxin users were defined as those with at least two outpatient service claims with the Anatomical Therapeutic Chemical (ATC) code of C01AA05 at any ambulatory record of hospitals or any one hospitalization with CRS listed among the claims diagnosis codes and with digoxin use.

### Outcome measures

Patients with ESRD on dialysis can apply for a catastrophic illness card in Taiwan. Cardholders are exempt from co-payment for the NHI. Patients with ESRD were defined as those who received a catastrophic illness card and required hemodialysis or peritoneal dialysis for at least 3 months (ICD-9-CM code 585). The CHF hospitalization and CAD hospitalization are defined as hospital admission after the index date with the main ICD-9-CM code with 428 and 410–414 separately. We linked the NHIRD to the Taiwanese National Death Registry to confirm the death. The person-years of follow-up of the patients were estimated from the index date to the study end-point date, censored by in-hospital death, loss to follow-up, withdrawal from the insurance system, or end date of 31 December 2010. The comorbidities included in our study were hypertension (ICD-9-CM codes 401–405), type 2 diabetes (ICD-9-CM code250), hyperlipidemia (ICD-9-CM code 272), cardiovascular disease (ICD-9-CM codes 410, 412, 428), cerebrovascular accident (ICD-9-CM codes 430–438), liver disease (ICD-9-CM codes 571–572, 456.0–456.2), and gout (ICD-9-CM code 274.x).

### Validation

We validated the ICD-9-CM codes for the identification of CRS by analyzing the medical records (charts) of 200 patients, who had CHF ICD-9-CM code 428; CKD ICD-9-CM code 585 from the inpatient and outpatient claims database between January 2008 and December 2010 in Kaohsiung Municipal Ta-Tung Hospital, which is a regional teaching hospital in Taiwan. The contents of this database were similar to those of the NHIRD. The clinical diagnosis of CHF and CKD was ascertained by physicians. Clinical diagnosis of CHF was determined by Framingham criteria, and clinical diagnosis of CKD was determined according to the eGFR < 60 ml/min/1.73 m^2^ for more than 3 months. Positive predictive values of both diseases were estimated. There are 184 cases that confirmed the diagnosis of CHF, and 193 cases confirmed the diagnosis of CKD. The positive predictive value (PPV) of CHF and CKD are 0.92 and 0.965, separately.

### Statistical analysis

An independent *t*-test, chi-square test, or Fisher's exact test was employed to compare the distribution of risk factors between the CRS and control cohorts. Cox proportional hazard regression analyses were conducted to calculate the crude and adjusted hazard ratios (aHRs) for the risk of clinical outcomes. Multiple Cox proportional hazard regression analyses were performed after adjustment for sex, age, and any history of hypertension, type 2 diabetes, hyperlipidemia, cerebral vascular accident, cardiovascular disease, liver disease, and gout. Kaplan–Meier curves were applied to estimate the probability of clinical outcomes onset, and the log-rank or Gehan–Breslow–Wilcoxon test was used to examine the differences among groups. Statistical analyses were performed using SAS 9.3 software (SAS Institute, Inc., Cary, NC, USA). Statistical significance was set at a *p*-value of < 0.05.

## Results

### Baseline characteristics of patients with CRS

We enrolled 9,509 patients in the CRS cohort. We excluded patients aged younger than 20 years (*n* = 83) or who did not have complete demographic data (*n* = 1, 018). There are 8,427 patients in the CRS cohort. In this cohort, there were 705 digoxin users. After being matched with a ratio of one to three, there were 2,115 digoxin non-users with no significant differences in age, gender, living regions, comorbidities, Charlson comorbidity index, and cardiovascular medications (except digoxin) with digoxin users (Table 1).

**Table 1:** Demographic characteristics between digoxin users and non-users in patients with CRS.

	**Digoxin users (*****n*** = **705)**		**Digoxin nonusers (*****n*** = **2,115)**		***p*-value**
**Age (Mean** ±**SD)**		**(**±**)**		**(**±**)**	
< 40	14	(2.0)	33	(1.6)	0.728
40–59	105	(14.9)	309	(14.6)	
≧60	586	(83.1)	1,773	(83.8)	
**Gender (%)**					
Female	326	(46.2)	975	(46.1)	0.948
Male	379	(53.8)	1,140	(53.9)	
**Region**					
Northern	286	(40.6)	834	(39.4)	0.849
Central	220	(31.2)	680	(32.2)	
Southern and eastern	199	(28.2)	601	(28.4)	
**Comorbidities (%)**					
Hypertension	181	(68.2)	1,417	(67.0)	0.547
Type 2 Diabetes	280	(39.7)	805	(38.1)	0.434
Hyperlipidemia	168	(23.8)	488	(23.1)	0.681
Cerebral vascular accident	61	(8.7)	181	(8.6)	0.938
Chronic liver disease	132	(18.7)	384	(18.2)	0.736
Gout	158	(22.4)	473	(22.4)	0.979
**Charlson comorbidity index**					
1	324	(46.0)	989	(46.8)	0.661
2	179	(25.3)	537	(25.4)	
≧3	202	(28.7)	589	(27.8)	
**Medication**					
ACEI	313	(44.4)	934	(44.2)	0.913
ARB	193	(27.4)	581	(27.5)	0.961
α-Blockers	126	(17.9)	375	(17.7)	0.932
β-Blockers	273	(38.7)	807	(38.2)	0.788
**Calcium channel blockers**					
Non-dihydropyridine	140	(19.9)	416	(19.7)	0.913
Dihydropyridine	301	(42.7)	894	(42.3)	0.843
Other antihypertensives	69	(9.8)	186	(8.8)	0.426

SD, standard deviation; ACEI, angiotensin-converting enzyme inhibitor; ARB, angiotensin II receptor blockers.

The difference between the two cohorts was estimated by an independent *t*-test or chi-square test.

### All-cause mortality of digoxin usage in CRS

**Figure 2:**
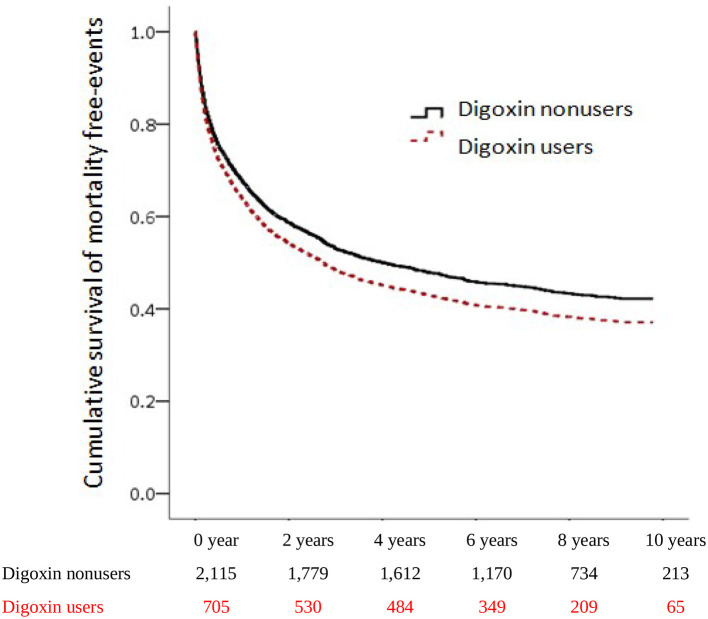
Cumulative hazards of all-cause mortality among patients with CRS.

To investigate the incidences of all-cause mortality in patients with digoxin usage in CRS, we examined the risk of death by using an adjusted Cox proportional hazard regression model. The mortality increased significantly from 50.17 per 1,000 patient-years of digoxin non-users to 64.95 per 1,000 patient-years of digoxin users. Patients receiving digoxin had the worse overall survival (aHR:1.26, [95% CI: 1.09–1.46], *P* = 0.002) (Table 2). This indicated that among patients with CRS, 26% of the risk of mortality increased in digoxin users compared with non-users. To explore the dosage effect of digoxin on death, we stratified the patients by defined daily dose (DDD). There was significantly higher mortality in the *0.26–0.75* DDD of digoxin users than in digoxin non-users (HR = 1.49; 95% CI = 1.23–1.82, *p*<*0.001*). Therefore, the *p for trend* was *0.013*. To validate the impact of digoxin on morality, we accomplished the Kaplan–Meier analysis for the adjusted cumulative hazards of mortality. Digoxin significantly increased the risk of death in CRS (*p* < 0.005) (Figure 2 and Table 3).

**Table 2:** Risk of mortality, CHF recurrence, CAD recurrence, and ESRD between digoxin users and non-users (*N* = 2,820).

	**Number of events**				**Digoxin users vs. non-users**			
	**Events**		**Events or death**		**Model I**		**Model II**	
	**No. cases**	**Per 1,000 PY**	**No. cases**	**Per 1,000 PY**	**aHR (95% CI)**	***p*-value**	**SHR (95%CI)**	***p*-value**
**Mortality**								
Comparison cohort	643	50.17			Ref.			
Digoxin cohort	250	64.95			1.26 (1.09–1.46)	0.002		
< 0.26 DDDs	81	62.40			1.20 (0.95–1.51)	0.130		
0.26–0.75 DDDs	117	76.62			1.49 (1.23–1.82)	< 0.001		
>0.75 DDDs	52	50.83			1.00 (0.75–1.32)	0.972		
*p for trend*						0.013		
**CHF hospitalization**								
Comparison cohort	1,142	143.92	1,351	170.26				
Digoxin cohort	390	174.63	468	209.55	1.15 (1.02–1.29)	0.018	1.17 (1.05–1.30)	0.004
< 0.26 DDDs	154	170.03	183	202.04	1.16 (0.98–1.37)	0.094	1.15 (0.99–1.34)	0.076
0.26–0.75 DDDs	113	178.80	136	215.19	1.14 (0.94–1.38)	0.188	1.18 (0.98–1.40)	0.074
>0.75 DDDs	123	176.83	149	214.21	1.15 (0.95–1.39)	0.142	1.19 1.01–1.41)	0.046
*p for trend*						0.036		0.006
**CAD hospitalization**								
Comparison cohort	931	107.76	1,247	144.33				
Digoxin cohort	267	96.60	399	144.36	0.88 (0.77–1.01)	0.074	0.99 (0.87–1.11)	0.919
< 0.26 DDDs	104	107.37	161	166.22	0.99 (0.81–1.22)	0.966	1.11 (0.94–1.31)	0.236
0.26–0.75 DDDs	76	65.36	113	97.18	0.92 (0.76–1.12)	0.426	1.02 (0.87–1.20)	0.823
>0.75 DDDs	87	137.56	125	197.65	0.67 (0.51–0.89)	0.005	0.79 (0.63–0.99)	0.048
*p for trend*						0.012		0.293
**ESRD**								
Comparison cohort	220	18.11	728	59.94				
Digoxin cohort	179	55.11	314	96.67	2.43 (1.94–3.04)	< 0.001	1.34 (1.16–1.54)	< 0.001
< 0.26 DDDs	48	43.99	86	78.83	2.64 (1.92–3.65)	< 0.001	1.32 (1.06–1.66)	0.015
0.26–0.75 DDDs	55	39.15	115	81.85	2.79 (2.06–3.79)	< 0.001	1.44 (1.18–1.75)	< 0.001
>0.75 DDDs	28	31.64	65	73.45	1.74 (1.16–2.60)	0.007	1.21 (0.94–1.57)	0.137
*p for trend*						< 0.001		< 0.001

Model I: adjusted Cox proportional hazard regression model.

Model II: competing risk analysis model.

Adjusted all comorbidities.

sHR, subdistribution hazard ratio; aHR, adjusted hazard ratio.

**Table 3:** Follow-up period of mortality, CHF hospitalization, CAD hospitalization, and ESRD between digoxin users and non-users.

	**Patient numbers**	**Mean (year)**	**SD (year)**	**Median (year)**	**Upper quadrants (year)**	**Lower quadrants (year)**
**Mortality**						
Comparison cohort	2,115	6.0597675	3.1388999	6.5452055	8.7863014	4.1342466
Digoxin cohort	705	5.4592014	3.3724509	5.9643836	8.4767123	2.0931507
**CHF hospitalization**						
Comparison cohort	2,115	3.7517135	3.4995028	2.6246575	6.8657534	0.4000000
Digoxin cohort	705	3.1678345	3.5093336	1.1068493	6.1780822	0.2027397
**ESRD**						
Comparison cohort	2,083	5.8864783	3.2303418	6.3479452	8.7178082	3.6328767
Digoxin cohort	657	5.1450053	3.4948127	5.7232877	8.1643836	1.2684932

### CHF hospitalization of digoxin usage in CRS

**Figure 3:**
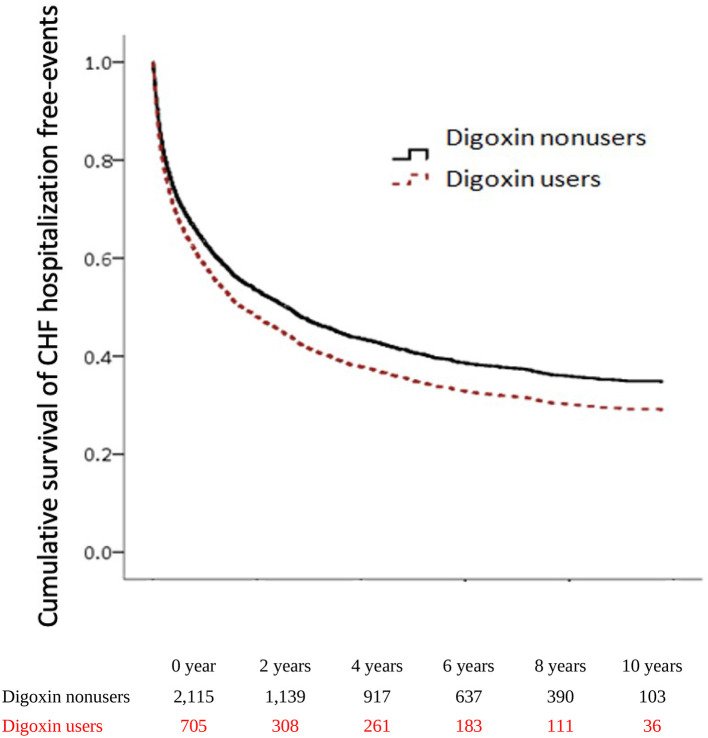
Cumulative hazards of CHF hospitalization among patients with CRS.

To explore the risk of CHF hospitalization with digoxin usage in patients with CRS, we executed both the adjusted Cox proportional hazard regression model (Model I) and the competing risk analysis model (Model II). In Model I, the CHF hospitalization was significantly higher in digoxin users than in non-users (aHR = 1.15; 95% CI = 1.02–1.29, *p* = *0.018*). In Model II, the digoxin increased the CHF hospitalization of patients with CRS (sHR = 1.17; 95% CI = 1.05–1.30, *p* = *0.004*). To examine the digoxin prescription for CHF hospitalization, we stratified the patients by DDD. In Model I, there was the *p for trend* = 0.036 of the digoxin usage on CHF hospitalization. In Model II, digoxin increased CHF hospitalization with >*0.75* DDD (sHR = 1.19; 95% CI = 1.23–1.82, *p*<*0.001*). Thus, the *p for trend* of the digoxin dosage effect on CHF hospitalization was *0.006*. To validate the impact of digoxin on CHF hospitalization, we executed the Kaplan–Meier analysis for the adjusted cumulative hazard. Digoxin meaningfully augmented the jeopardy of CHF hospitalization in CRS (*p* < 0.005) (Figure 3 and Table 3).

### CAD hospitalization of digoxin usage in CRS

To assess the risk of CAD hospitalization with digoxin use in patients with CRS, we performed both Models I and II. In Model I, CAD hospitalization was significantly lower in the >*0.75DDD* subgroup of digoxin users (aHR = 0.67; 95% CI = 0.51–0.89, *p* = *0.005*). Therefore, the *p for trend* of the digoxin dosage effect on CAD hospitalization was *0.012*. In Model II, digoxin prevented CAD hospitalization in the >*0.75DDD* (sHR = 0.79; 95% CI = 0.63–0.99, *p* = *0.048*).

### ESRD of digoxin usage in CRS

**Figure 4:**
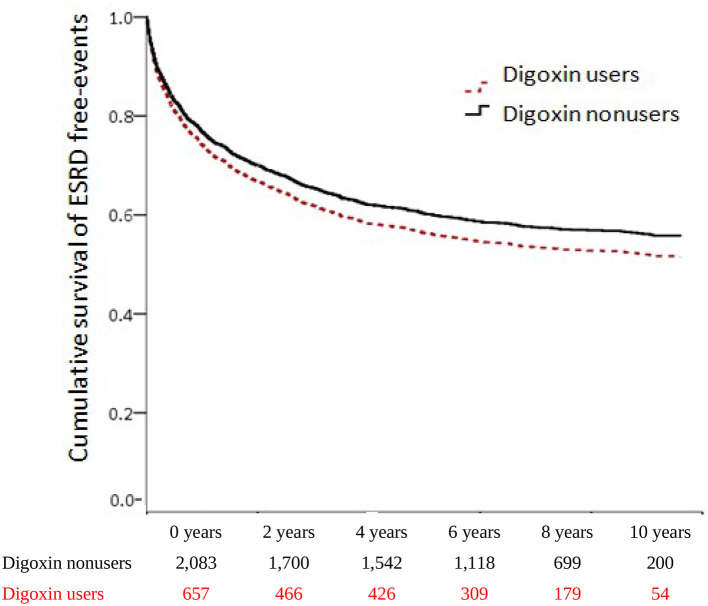
Cumulative hazards of ESRD among patients with CRS.

To evaluate the digoxin effect on renal function progression in patients with CRS, we executed both Model I and Model II analyses. In Model I, digoxin usage ominously augmented the risk of ESRD (aHR = 2.43; 95% CI = 1.94–3.04, *p* < 0.001). In Model II, patients with CRS who took digoxin upsurged the threat of ESRD (sHR = 1.34; 95% CI = 1.16–1.54, *p* < 0.001). To explore the digoxin dosage on kidney failure for patients with CRS, we stratified patients by digoxin DDD. In Model I, digoxin amplified incident ESRD with all tertials of DDD (< 0.26 DDD, aHR = 2.64; 95% CI = 1.92–3.65, *p* < 0.001; 0.26–0.75 DDD, aHR = 2.79; 95% CI = 2.06–3.79, *p* < 0.001; >0.75 DDD, aHR = 1.74; 95% CI = 1.16–2.60, *p* < 0.001). Then, the *p for trend* < 0.001. In Model II, ESRD occurrence magnified with the DDD < 0.26 and 0.26–0.75 DDD of digoxin usage in patients with CRS (sHR = 1.32; 95% CI = 1.06–1.66, *p* = 0.015; sHR = 1.44; 95% CI = 1.18–1.75, *p* < 0.001). There was a significant *p for trend* < 0.001 of the digoxin usage on ESRD incidence. To confirm the effect of digoxin on ESRD existence, we accomplished the Kaplan–Meier analysis for the adjusted cumulative hazard. Digoxin knowingly increased the danger of renal function decline with entering ESRD in CRS (*p* < 0.005) (Figure 4 and Table 3).

### Sensitivity analysis

In sensitivity analyses, we found that the estimated effects of digoxin use were similar, except for CAD recurrence when we changed the entry and observation periods. The results of sensitivity analyses are represented in Table 4. These results indicated that our findings were robust.

**Table 4:** Risk of mortality, CHF recurrence, CAD recurrence, and ESRD between digoxin users and non-users from 2000 to 2003 year (*N* = 1,806).

	**No. of events**				**Digoxin users vs. non-users**			
	**Events**		**Events or death**		**Model I**		**Model II**	
	**No. cases**	**(%)**	**No. cases**	**(%)**	**aHR (95% CI)**	***p*-value**	**SHR (95%CI)**	***p*-value**
**Mortality**								
Comparison cohort	436	31.9			Ref.			
Digoxin cohort	170	38.7			1.35 (1.13–1.62)	0.001		
**CHF hospitalization**								
Comparison cohort	738	54.0	883	64.6	Ref.		Ref.	
Digoxin cohort	254	57.9	303	79.0	1.29 (1.12–1.49)	0.001	1.29 (1.13–1.47)	< 0.001
**CAD hospitalization**								
Comparison cohort	602	44.0	816	59.7	Ref.		Ref.	
Digoxin cohort	176	40.1	259	59.0	0.98 (0.83–1.16)	0.828	1.07 (0.93–1.24)	0.323
**ESRD**								
Comparison cohort	116	8.6	456	35.9	Ref.		Ref.	
Digoxin cohort	87	21.5	173	42.8	2.65 (2.00–3.51)	< 0.001	1.42 (1.19–1.69)	< 0.001

Model I: adjusted cox proportional hazard regression model.

Model II: competing risk analysis model.

Adjusted all comorbidities.

sHR, subdistribution hazard ratio; aHR, adjusted hazard ratio.

## Discussion

We investigated the association of clinical outcomes between digoxin users and non-users in patients with CRS. We demonstrated that prescription digoxin in patients with CRS increased the threat of all-cause mortality, CHF recurrence, and ESRD.

In the general population, despite the ideal medications with angiotensin-converting enzyme inhibitors (ACEI), beta-blockers, and mineralocorticoid receptor antagonists (MRAs), the treatment for patients with heart failure remain unsatisfied due to hypotension, hyperkalemia, and renal function deterioration. Digoxin is still one of the most common prescriptions for the treatment of CVD. Due to its unique inotropic and chronotropic properties, it is used for the treatment of heart failure and atrial fibrillation (20, 21). Digoxin inhibits sodium–potassium adenosine triphosphatase (Na+/K+-ATPase), and it increases the intracellular content of sodium and calcium ions in myocytes to augment heart contractility. However, digoxin has a narrow therapeutic window and accordingly needs to modify doses based on age, weight, and renal function with a close monitor. The renal clearance of digoxin declines linearly with the progression of eGFR, and therefore any disturbance of renal function may affect digoxin efficacy and rise toxicity. Patients with CRS are in double jeopardy of worsening heart and kidney function progression. More evidence-based treatments for patients with CRS are a necessity for decision-making initiation, drug dose titration, and therapy discontinuation.

Few trials have been conducted to examine whether it is safe to prescribe digoxin in patients with CRS, which is prone to worse renal function and may enter ESRD for long-term renal replacement therapy. In the Digitalis Investigation Group (DIG) trial, improvement of renal function (defined as ?20% increase in eGFR post-randomization of digoxin prescription) occurred in 15% of the study population (overall eGFR = 70.0± 21.7 ml/min/1.73 m^2^) and was more common in patients receiving digoxin treatment (*p* = 0.02) (22). Then, in the group of improvement of renal function, digoxin usage was associated with improvement of free hospitalization free survival (adjusted HR = 0.49, 95% CI: = 0.3–0.8, *p* = 0.006, p interaction = 0.026). The secondary analysis of the DIG trial categorized by eGFR was conducted by Shlipak et al. (23). The all-cause mortality significantly increased with the decline of eGFR (GFR >60, 31% mortality; GFR 30–60, 46% mortality; GFR < 30, 62% mortality; *p* < 0.001). Thus, the effect of digoxin on all-cause mortality varied from 0.93 (GFR < 30) to 1.01 (GFR> 60), and for the combined outcome of death or heart failure hospitalization from 0.77 (GFR < 30) to 0.84 (GFR 30 to 60; *P* > 0.10 in both cases for interaction). The digoxin efficacy did not differ by level of eGFR (*p* = 0.19 for interaction). However, in the study of Chan et al., which analyzed the database of Fresenius Medical Care North America (FMCNA) facility among 120,864 incident hemodialysis patients, digoxin usage was associated with a 28% increased risk for death (HR 1.28; 95% CI: 1.25–1.31) (17). Thus, the all-cause mortality was significantly associated with increased serum digoxin level (HR 1.19 per ng/ml increase; 95% confidence interval 1.05–1.35). A study from Canada explored the risk of digoxin toxicity in older patients with CKD by using a population-based study (24). Starting digoxin at >0.125 vs. ≤ 0.125 mg/day was associated with a higher 90-day risk of hospital admission, or an emergency department (ED) visit with toxicity: 149 vs. 33 events per 1,000 person-years (weighted HR (wHR), 5.75 [95% CI: 4.00–8.27]). Our study has tried to fulfill the gap to investigate the clinical outcomes of the prescription of digoxin in patients with CRS. Then, the result of our study is in accordance with some of the previous studies that there were significant *p* for trends of increasing risks of all-cause mortality, heart failure recurrence, and ESRD in increasing dosage of digoxin among patients with CRS. Prescription the large dosage of digoxin is more hazardous than low doses.

Due to the narrow therapeutic range of digoxin, digoxin toxicity is not uncommon. Digoxin is a toxic substance with cardiotoxic activity (25). In animal studies, digoxin toxicity can occur during long-term therapy, like what occurs in human medicine (26). The incidence of digoxin toxicity raises from 1% in patients with greater than age 40 to 3% in patients over age 85 (27). In the study by Wei et al., which analyzed the US Food and Drug Administration Adverse Event Reporting System from 1986 to 2019, the most frequently reported adverse events were cardiac (bradycardia, cardiac arrest, and hypotension) and non-cardiac (nausea and hyperkalemia) (28). Although digoxin targets Na+/K+-ATPase, it may poison the Na-K transporter and block the AV node with increasing vagal tone. The following upsurge in intracellular sodium leads to a rise in intracellular calcium by reducing calcium excretion through the sodium-calcium cation exchanger (29). Elevated intracellular calcium due to Na-K transporter poisoning and AV node blockade due to augmented vagal tone are major causes of digoxin toxicity. The first leads to increased automaticity and inotropy, and the latter leads to decreased dromotrophy. With the decline of renal clearance of digoxin with CKD, the digoxin poisonousness may rise and lead to worse clinical outcomes. Monitoring cardiac function, electrolyte imbalance, and renal function closely in patients with CRS may be able to prevent toxicity from happening.

Prescription of patients with CRS is always a therapeutic challenge for clinicians. Because of concerns about a rise in serum creatinine and potassium levels, physicians may hesitate to prescribe angiotensin-converting enzyme inhibitor (ACEI)s/angiotensin II receptor blockers (ARB)s in patients with CRS. Diuretics treatment is the choice to reduce symptoms of volume overload and short-time decrease estimate glomerular filtration rate (eGFR) with a better prognosis in CRS (30). However, diuretic resistance may happen and indicate a worse outcome (31). In the condition of intravascular volume depletion and poor cardiac function with renal dysfunction, chronotropic agents may help to improve cardiac output and reverse the worsening of heart and kidney interaction. Our analysis tried to concentrate on the cardiorenal effect as a whole and found that digoxin use might increase the incidence of ESRD and CHF hospitalization in patients with CRS. There was a significant *p for trends* of increasing risks of mortality, CHF hospitalization, and ESRD in increasing dosage of digoxin among patients with CRS. Therapeutic drug monitoring of its trough plasmatic concentration may be useful to prevent toxicity (32).

This present study has some limitations. First, the NHI database does not include laboratory data such as potassium and creatinine levels to further analyze the proportion of the type of CRS, and the effect of hypokalemia and eGFR status. Second, owing to the claim data, we defined the date of the first medical claim with the corresponding ICD-9-CM code as the date of diagnosis and the age at diagnosis. The underestimation of the prevalence of CRS is easily possible. Third, the sample sizes of some subgroups were comparably small due to stratification, which lowered the statistical power of the study. Fourth, this study design is a retrospective cohort study and does not seek to investigate causal inferences. Fifth, this study excluded those with a loss of demographic data, and this loss of data may not be random.

## Conclusion

Our data provide the first evidence with a human nationwide cohort of the association of digoxin prescription with clinical outcomes in CRS. In conclusion, digoxin should be prescribed with caution to patients with CRS. We reinforce that it is necessary to frequently monitor serum-level to achieve a narrow therapeutic window.

## Data availability statement

The original contributions presented in the study are included in the article/supplementary material, further inquiries can be directed to the corresponding author.

## Ethics statement

The studies involving human participants were reviewed and approved by Kaohsiung Medical University Hospital. Written informed consent for participation was not required for this study in accordance with the national legislation and the institutional requirements.

## Author contributions

K-TC, Y-HC, and HL conceived the project and designed the experiments. Y-HC and Y-TW designed the statistical model. H-FK, S-WN, and I-CK executed the computational framework. Z-HW, C-CH, and YC contributed to data interpretation and modification of the experiment design. The manuscript was composed by K-TC and HL, with inputs from all authors. L-JY and J-MC provided overall supervision of the project. All authors contributed to the article and approved the submitted version.
